# Supplementary material for: An Italian Real-World Study Highlights the Importance of Some Clinicopathological Characteristics Useful in Identifying Metastatic Breast Cancer Patients Resistant to CDK4/6 Inhibitors and Hormone Therapy
Source: Biomedicines. 2024 Feb 22;12(3):498. doi: 10.3390/biomedicines12030498 (PMC10968497; doi:10.3390/biomedicines12030498)
Supplement: Supplementary file 1 [file biomedicines-12-00498-s001.zip › biomedicines-2762480-supplementary.pdf]

Supplementary table S1: Baseline characteristics of patients with a BMI less or equal 25 kg/m2 and greater than 25 kg/m2 in the overall population and stratified by treatment group

	Total, N° (%) (N = 177)			CDK 4/6 Inhibitors + Letrozole, N° (%) (N = 66)			CDK 4/6 Inhibitors + Fulvestrant, N° (%) (N = 111)		
**Baseline characteristics**	**BMI≤25 kg/m2 (n = 90)**	**BMI≥25 kg/m2 (n = 87)**	*p-value*	**BMI≤25 kg/m2 (n = 37)**	**BMI≥25 kg/m2 (n = 29)**	*p-value*	**BMI≤25 kg/m2 (n = 53)**	**BMI≥25 kg/m2 (n = 58)**	*p-value*
Age									
Median (min -max)	60 (38-92)	65 (39-84)	0.306	60 (43-87)	63 (40-83)	0.596	59 (38-92)	66 (39-84)	0.346
<=65 y	59 (65.6 %)	44 (50.6 %)	0.043	23 (62.2 %)	16 (55.2 %)	0.566	36 (67.9 %)	28 (48.3 %)	0.036
>65 y	31 (34.4 %)	43 (49.4 %)		14 (37.8 %)	13 (44.8 %)		17 (32.1 %)	30 (51.7 %)	
Menopausal status									
Postmenopause	76 (84.4 %)	76 (87.4 %)	0.578	33 (89.2 %)	26 (89.7 %)	0.951	43 (81.1 %)	50 (86.2 %)	0.469
Pre - or perimenopause	14 (15.6 %)	11 (12.6 %)		4 (10.8 %)	3 (10.3 %)		10 (18.9 %)	8 (13.8 %)	
Previous HT									
No	25 (27.8 %)	17 (19.5 %)	0.198	16 (43.2 %)	14 (48.3 %)	0.684	9 (17.0 %)	3 (5.2 %)	0.045
Yes	65 (72.2 %)	70 (80.5 %)		21 (56.8 %)	15 (51.7 %)		44 (83.0 %)	55 (94.8 %)	
Prior Aromatase Inhibitor									
No	42 (46.7 %)	33 (37.9 %)	0.240	24 (64.9 %)	18 (62.1 %)	0.815	18 (34.0 %)	15 (25.9 %)	0.315
Yes	48 (53.3 %)	54 (62.1 %)		13 (35.1 %)	11 (37.9 %)		35 (66.0 %)	43 (74.1 %)	
Prior adjuvant chemotherapy									
No	56 (62.2 %)	50 (57.5 %)	0.519	24 (64.9 %)	21 (72.4 %)	0.513	32 (60.4 %)	29 (50.0 %)	0.272
Yes	34 (37.8 %)	37 (42.5 %)		13 (35.1 %)	8 (27.6 %)		21 (39.6 %)	29 (50.0 %)	
ER status (Positive if value ≥ 10)									
Positive	88 (97.8 %)	86 (98.9 %)	0.580	36 (97.3 %)	28 (96.6 %)	0.861	52 (98.1 %)	58 (100 %)	0.293
Negative	2 (2.2 %)	1 (1.1 %)		1 (2.7 %)	1 (3.4 %)		1 (1.9 %)	0 (0.0 %)	
PgR status (Positive if value ≥ 20)									
Positive	66 (73.3 %)	73 (83.9 %)	0.087	31 (83.8 %)	25 (86.2 %)	0.785	35 (66.0 %)	48 (82.8 %)	0.043
Negative	24 (26.7 %)	14 (16.1 %)		6 (16.2 %)	4 (13.8 %)		18 (34.0 %)	10 (17.2 %)	
Previous lines									
First line	49 (54.4 %)	46 (52.9 %)	0.834	36 (97.3 %)	29 (100.0 %)	0.372	13 (24.5 %)	17 (29.3 %)	0.571
≥ 2 lines	41 (45.6 %)	41 (47.1 %)		1 (2.7 %)	0 (0.0 %)		40 (75.5 %)	41 (70.7 %)	
Metastatic site									
Bone only	21 (23.3 %)	24 (27.6 %)	0.764	7 (18.9 %)	7 (24.1 %)	0.716	14 (26.4 %)	17 (29.3 %)	0.478
Visceral	36 (40.0 %)	31 (35.6 %)		11 (29.7 %)	10 (34.5 %)		25 (47.2 %)	21 (36.2 %)	
Other	33 (36.7 %)	32 (36.8 %)		19 (51.4 %)	12 (41.4 %)		14 (26.4 %)	20 (34.5 %)	
Organ involved, N°									
1	39 (43.3 %)	36 (41.4 %)	0.297	17 (45.9 %)	11 (37.9 %)	0.687	22 (41.5 %)	25 (43.1 %)	0.066
2	33 (36.7 %)	40 (46.0 %)		16 (43.2 %)	13 (44.8 %)		17 (32.1 %)	27 (46.6 %)	
≥ 3	18 (20.0 %)	11 (12.6 %)		4 (10.8 %)	5 (17.2 %)		14 (26.4 %)	6 (10.3 %)	
Neutropenia									
Absent	42 (46.7 %)	55 (63.2 %)	0.027	16 (43.2 %)	19 (65.5 %)	0.072	26 (49.1 %)	36 (62.1 %)	0.168
Present	48 (53.3 %)	32 (36.8 %)		21 (56.8 %)	10 (34.5 %)		27 (50.9 %)	22 (37.9 %)	
Ki67 (%)									
Median (min -max)	20 (2-80)	18 (0-65)	0.351	15 (5-45)	15 (5-45)	0.532	23 (2-80)	20 (0-65)	0.341
<20%	39 (43.3 %)	46 (52.9 %)	0.204	24 (64.9 %)	20 (69.0 %)	0.726	15 (28.3 %)	26 (44.8 %)	0.072
≥ 20%	51 (56.7 %)	41 (47.1 %)		13 (35.1 %)	9 (31.0 %)		38 (71.7 %)	32 (55.2 %)	

Supplementary table S2: Multivariable Cox PH model - Progression risk factors

	HR (95% CI)		*p-value*
**Previous Line (≥2)**	1.780	(1.224-2.588)	0.003
**N. metastasis (2+)**	2.105	(1.350-3.277)	0.001

Supplementary table S3: Patients who presented neutropenia

**Neutropenia**									
	N (%)	N (%)		N (%)	N (%)	N (%)	N (%)	N (%)	N (%)
**Absent**	42 (46.7%)	55 (63.2%)	0.027	16 (43.2%)	19 (65.5%)	0.072	26 (49.1%)	36 (62.1%)	0.168
**Present**	48 (53.3%)	32 (36.8%)		21 (56.8%)	10 (34.5%)		27 (50.9%)	22 (37.9%)	
